# Insulin and Glucagon Regulate Pancreatic α-Cell Proliferation

**DOI:** 10.1371/journal.pone.0016096

**Published:** 2011-01-25

**Authors:** Zhuo Liu, Wook Kim, Zhike Chen, Yu-Kyong Shin, Olga D. Carlson, Jennifer L. Fiori, Li Xin, Joshua K. Napora, Ryan Short, Juliana O. Odetunde, Qizong Lao, Josephine M. Egan

**Affiliations:** National Institute on Aging, National Institutes of Health, Baltimore, Maryland, United States of America; Mayo Clinic College of Medicine, United States

## Abstract

Type 2 diabetes mellitus (T2DM) results from insulin resistance and β-cell dysfunction, in the setting of hyperglucagonemia. Glucagon is a 29 amino acid peptide hormone, which is secreted from pancreatic α cells: excessively high circulating levels of glucagon lead to excessive hepatic glucose output. We investigated if α-cell numbers increase in T2DM and what factor (s) regulate α-cell turnover. Lepr^db^/Lepr^db^ (db/db) mice were used as a T2DM model and αTC1 cells were used to study potential α-cell trophic factors. Here, we demonstrate that in db/db mice α-cell number and plasma glucagon levels increased as diabetes progressed. Insulin treatment (EC50 = 2 nM) of α cells significantly increased α-cell proliferation in a concentration-dependent manner compared to non-insulin-treated α cells. Insulin up-regulated α-cell proliferation through the IR/IRS2/AKT/mTOR signaling pathway, and increased insulin-mediated proliferation was prevented by pretreatment with rapamycin, a specific mTOR inhibitor. GcgR antagonism resulted in reduced rates of cell proliferation in αTC1 cells. In addition, blockade of GcgRs in db/db mice improved glucose homeostasis, lessened α-cell proliferation, and increased intra-islet insulin content in β cells in db/db mice. These studies illustrate that pancreatic α-cell proliferation increases as diabetes develops, resulting in elevated plasma glucagon levels, and both insulin and glucagon are trophic factors to α-cells. Our current findings suggest that new therapeutic strategies for the treatment of T2DM may include targeting α cells and glucagon.

## Introduction

Type 2 diabetes mellitus (T2DM) is considered to be a direct consequence of insulin resistance and β-cell dysfunction. Pancreatic α cells and their secretary product, glucagon, are often overlooked even though a “bihormonal abnormality” theory of T2DM pathology was proposed as long ago as 35 years [1]. Clinical studies show that increased fasting glucagon levels and lack of suppression of postprandial glucagon secretion are responsible for the increased glucose levels observed in T2DM [1]–[6]. Long-term hyperglucagonemia, as seen with glucagonomas, also causes a T2DM phenotype, and glucagonomas in mice cause a metabolic phenotype characteristic of T2DM [7]. It is poorly understood why plasma glucagon levels are elevated in T2DM patients. The major targets of glucagon are hepatocytes on which glucagon receptors (GcgRs) are prevalent, but GcgRs are also expressed on β cells [8] and GcgR null mice are resistant to β-cell loss and hyperglycemia [9]. There is also some evidence that glucagon directly regulates α-cell activity. GcgRs are expressed on α cells and glucagon was shown to stimulate exocytosis from mouse and rat α cells [10]. Additionally, glutamate co-secreted from α-cell secretory granules with glucagon directly causes additional α-cell exocytosis through glutamate receptor activation [11].

To date, the dysfunction of glucagon secretion in diabetes is vaguely considered to result from defective glucose sensing and insulin resistance in liver and muscle [12]. Recently, several papers have focused on the signaling pathways in α cells that regulate glucagon secretion under physiological conditions [10], [11], [13], [14]. Regulation of α-cell function and turnover in the diabetic state deserves more investigation so as to better design therapeutic strategies and while much work has been done on understanding how pancreatic β-cell function and proliferation are regulated, factors that regulate α-cell proliferation are largely ignored. We hypothesize that not only increased glucagon secretion but also increased α-cell proliferation is responsible for the elevated glucagon levels that occur in T2DM. There are data pointing to increased α-cell numbers in T2DM [15] and therefore we sought to determine if, in fact, α-cell numbers increase during the development of T2DM, and what factors, especially if any intra-islet factors, control pancreatic α-cell proliferation. There are several candidate islet factors that may be involved in α-cell proliferation: (1) glucagon itself, because it regulates its own secretion, (2) insulin, because it directly regulates glucagon secretion through insulin receptors (IR) on α cells [13], it regulates β-cell proliferation [16]–[18], and therefore may also regulate α-cell proliferation.

In recent years, it has been reported that blocking glucagon receptors improves glucose homeostasis [19]–[21]. Yet, targeting glucagon as a therapy for T2DM is not well developed, at least compared with the overwhelming information and attempts at modulating insulin receptor function. In this study, we used cell lines and animal models of diabetes. We found that α-cell numbers increased as blood glucose levels increase, insulin regulates α-cell proliferation by signaling through mTOR and glucagon receptor antagonism is beneficial to both α and β cells.

## Materials and Methods

### Animal study

Lepr^db^/Lepr^db^ (db/db) and Lepr^db^/^+^ (non-diabetic) mice (male) were from Jackson laboratories and were housed in the NIH/NIA mouse barrier facility with access to standard chow and water ad libitum. Over time, db/db mice displayed typical phenotypes of obesity, polyuria and hyperglycemia. Streptozotoxin (50 mg/kg) was administrated by daily intraperitoneal (i.p.) injection into 3-month-old CD1 mice for 5 days (n = 5). One month later, one mouse having died, their pancreata were collected for morphological analyses. Some db/db and control mice were given 20 µl (50 mM) of GcgR antagonist II (Calbiochem) (control n = 4, treated n = 5) orally once daily for 17 days. All animal testing procedures were approved by the Animal Care and Use Committee of the National Institute on Aging. The ID number of the animal study is 156 JME.

### Cell culture and reagents

The glucagon-producing αTC1 and insulin-producing mouse cell lines were grown in DMEM medium (Invitrogen) containing 4.5 g/L D-glucose, supplemented with fetal bovine serum (10% for αTC1 and 15% for MIN6), 1% L-Glutamine, 1% streptomycin/penicillin. CHO cells were grown in F-12 medium (Invitrogen) containing L-glutamine, supplemented with 10% fetal bovine serum and 1% streptomycin/penicillin.

### Transwell cell co-culture and cell proliferation

αTC1 cells were co-cultured with MIN6, αTC1, CHO cells or medium for 7 days using HTS Transwell-96 Tissue Culture Systems (Corning). Cells were also stimulated as follows for 72 hours: 14 µM GcgR Antagonist II, 20 ng/ml rapamycin (a kind gift from Dr. Paritosh Ghosh), recombinant insulin (Upstate Biotechnology), concentration and time points are indicated in figures or legends. The cells were grown from 3–5 days before treatment. Cell proliferation was assayed using The CellTiter 96 AQ_ueous_ One Solution Cell Proliferation Assay (Promega). This assay is a colorimetric method for determining the number of viable cells in proliferation [22]. After 1 hour at 37°C in a humidified, 5% CO_2_ atmosphere, the absorbance at 490 nm was recorded using an ELISA plate reader (Molecular Devices).

### Propidium iodide staining for DNA cell cycle analysis

αTC1 cells were serum starved for 5 h, followed by treatment with insulin (2 nM) in the absence or presence of rapamycin (20 ng/ml) for 24 h. The medium was collected and transferred into a 15 ml conical tube on ice. Cells were washed with PBS, trypsinized, and collected into conical tubes containing medium. After centrifugation, the cell pellets were gently resuspended in 300 µl of PBS to break up clumps and, while vortexing, 10 ml of ice cold 70% ethanol was added before storage at −20°C overnight. The following day, cells were centrifuged, washed with PBS, and 1 ml of 50 µg/ml propidium iodide solution (Calbiochem) containing 1 mg/ml RNAse (Ambion) was added. The cells were placed in the dark for 1 h prior to analysis using a BD FACS Calibur flow cytometer.

### Plasma glucose and hormone assay

We quantified plasma glucose levels using a glucose analyzer (Beckman Instruments). We measured plasma glucagon by RIA (Linco Research) and insulin by ELISA (Alpco Diagnostics).

### Immunofluorescence

Cell lines were cultured on glass slides for 48 hours before staining. Mouse pancreata were fixed in 4% paraformaldehyde for 2 hours, followed by immersion in 20% sucrose overnight at 4°C and then frozen. Sections (7 µm) were cut on a cryostat. Sections of human pancreas tissue were acquired from Histological Control Systems Inc (catalog # cs039-SAMPLE). Antigen retrieval (Citra solution, Biogenes) was performed on tissue sections before staining. Thereafter, sections were incubated overnight with the following primary antibodies: mouse anti-insulin (1∶200; Sigma), guinea pig anti-glucagon (1∶2000; Sigma) and rabbit anti-p-insulin Rβ (1∶100; Santa Cruz). Cell nuclei were stained with topro-3 (1∶2000; Invitrogen). The antigens were visualized using appropriate secondary antibody conjugated with Fluorescein FITC and cyanine Cy5 (1∶1000; Jackson ImmunoResearch Laboratories). All images were digitally acquired and were not further processed. Sections were viewed at 40× and 63× magnification. Digital images were compiled using Zeiss LSM image browser. Only brightness and contrast were adjusted.

### Immunoblot analyses

For western blot analyses, 20 µg of protein was separated by 4–12% Tris-Glycine gels (Invitrogen) electrophoresis and electroblotted onto PVDF filters (Invitrogen).

For protein detection, the following primary antibodies were used: mouse anti-IRβ (1∶1000, Santa Cruz), rabbit anti-IGF-1R (1∶1000, Cell Signaling), rabbit anti-p-insulin Rβ (1∶1000; Santa Cruz), rabbit anti-p-IRS1/2(Tyr612) (1∶1000, Santa Cruz), rabbit anti-p-PDK1 (Ser241) (1∶2000, Cell Signaling), rabbit anti-p-AKT (Ser473) (1∶1000, Cell Signaling), rabbit anti-AKT (1∶1000, Cell Signaling), rabbit anti-p-FOXO1 (Ser256) (1∶2000, Abcam), mouse p27 (1∶2500, BD), rabbit anti-p-mTOR (Ser2448) (1∶1000, Cell Signaling), rabbit anti-mTOR (1∶1000, Cell Signaling), β-Actin (1∶2000, Cell signaling) and GAPDH (1∶2000, Abcam) were used as internal control. HRP-linked (Abcam) secondary antibodies were visualized using ECL (GE Healthcare). For immunoprecipitation assays, αTC1 cells were lysed with ice-cold RIPA buffer. Cell lysate was then subjected to immunoprecipitation with anti-IRS1 (1∶1000, Santa Cruz) or anti-IRS2 (1∶1000, Santa Cruz) and immunoblotted with anti-p-IRS1/2 (Tyr612) (1∶1000, Santa Cruz).

### DAB staining and pancreatic α cell number counting

Mice pancreas frozen sections was stained with guinea pig anti-glucagon (1∶1000; Sigma). DAB (3,3-diaminobenzidine) staining was performed using ultravision mouse tissue detection system kit (labvision) following IHC slide staining DAB LABVISION PROTOCOL.

Quantification of DAB stained pancreatic images were performed in Matlab (Mathworks) (http://www.mathworks.com), a computer program used for technical computing, using novel software in conjunction with the image processing toolbox.

### Statistical analysis

Data are expressed as means ± SEM, and statistical significance was tested by Student's t test. Asterisks indicate statistical significance as follows: *, *P*<0.05; **; *P*<0.005, ***; *P*<0.0005.

## Results

### 1. Elevated plasma glucose levels in diabetic mice is accompanied by increased pancreatic α-cell number

**Figure 1:**
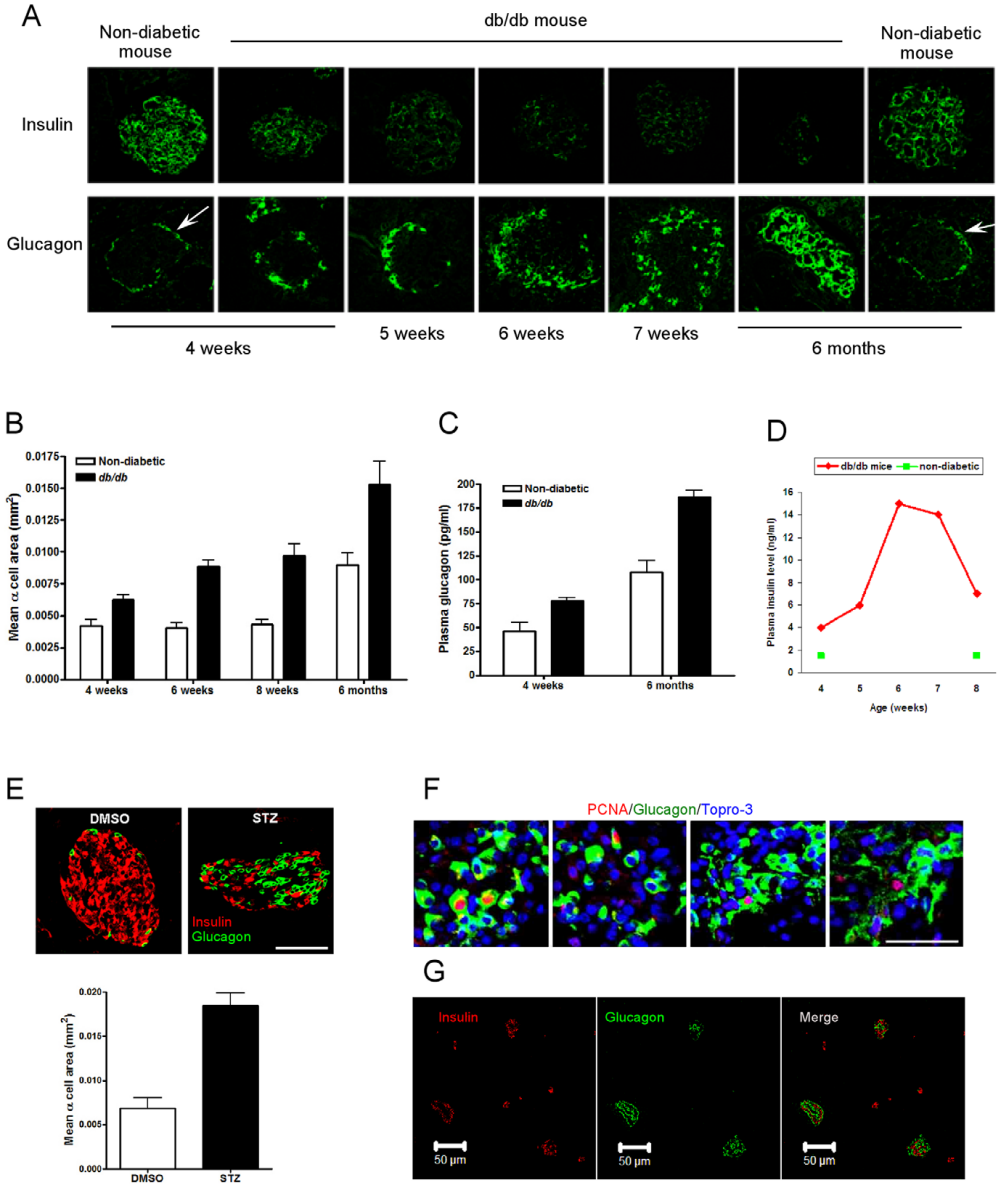
α-cell numbers are increased in db/db and streptozotocin (STZ)-treated mice. (A) Immunofluorescent staining of insulin and glucagon in mice at ages shown. Glucagon-positive cells are on the rim of islets in non-diabetic mice (arrows). (B) Mean glucagon-positive area in islets over time, in non-diabetic and db/db mice. (C) Random plasma glucagon levels in non-diabetic and db/db mice. (D) Random plasma insulin levels, over time, in db/db and non-diabetic mice. (E) Immunofluorescent staining of insulin and glucagon and quantification of glucagon-positive area in islets of STZ-treated CD1 mice. (F) PCNA-positive nuclei in glucagon-positive cells in islets from four different db/db mice. (G) Immunofluorescent staining of insulin and glucagon in human pancreatic paraffin sections, demonstrating lack of stereotypy in the distribution of α and β cells in human islets.

We evaluated the islets of db/db mice, a well-characterized mouse model of T2DM, and non-diabetic heterozygous Lepr^db^/^+^ mice, as blood glucose homeostasis worsens over time in the animals. Using immunohistochemical techniques, it can be seen the number of α cells obviously increased while insulin staining decreased over time (Figure 1A) as blood glucose rose (from normal at 4 weeks to >400 mg/dl at 7 weeks) in db/db mice. In conjunction, db/db mice had an increased α-cell area compared with non-diabetic mice and α-cell area increased over time (Figure 1B). Consistently, plasma glucagon levels were higher in db/db mice compared with non-diabetic mice (Figure 1C). The decreased insulin staining did not result in decreased plasma insulin levels when compared to non-diabetic animals, and must be at least in part due to degranulation of β cells because fasting plasma levels of insulin were extremely elevated, compared to non-diabetic mice (Figure 1D). In non-diabetic littermates there was no change in the islet morphology out to 6 months of age (Figure 1A). To confirm increasing α-cell numbers in another model of diabetes, we induced diabetes by giving streptozotocin (STZ) daily for 5 days to CD1 mice. Blood glucose was >500 mg/dl, 4 weeks after STZ. This particular mouse strain under normal conditions has few α cells compared to β-cell numbers in islets (96∶4 ratio) and therefore increasing α-cell numbers, α-cell area and dispersal of α cells thoughout the islets became obvious as a result of the STZ treatment (Figure 1E). The increased α-cell number is as a result of increased proliferation because numerous PCNA-positive α cells are evident in every islet from db/db mice at 7 weeks of age (four islets from four different mice are shown (Figure 1F), and no PCNA-positive α-cell could be found in non-diabetic mice. We had previously observed an increase in α-cell numbers in a Huntington's Disease mouse model that also develops diabetes [23]. We next investigated if α-cell proliferation is regulated by islet hormones.

### 2. Up-regulation of pancreatic α cell proliferation

#### 2.1 Co-culture of β cells with α cells increases α-cell proliferation

**Figure 2:**
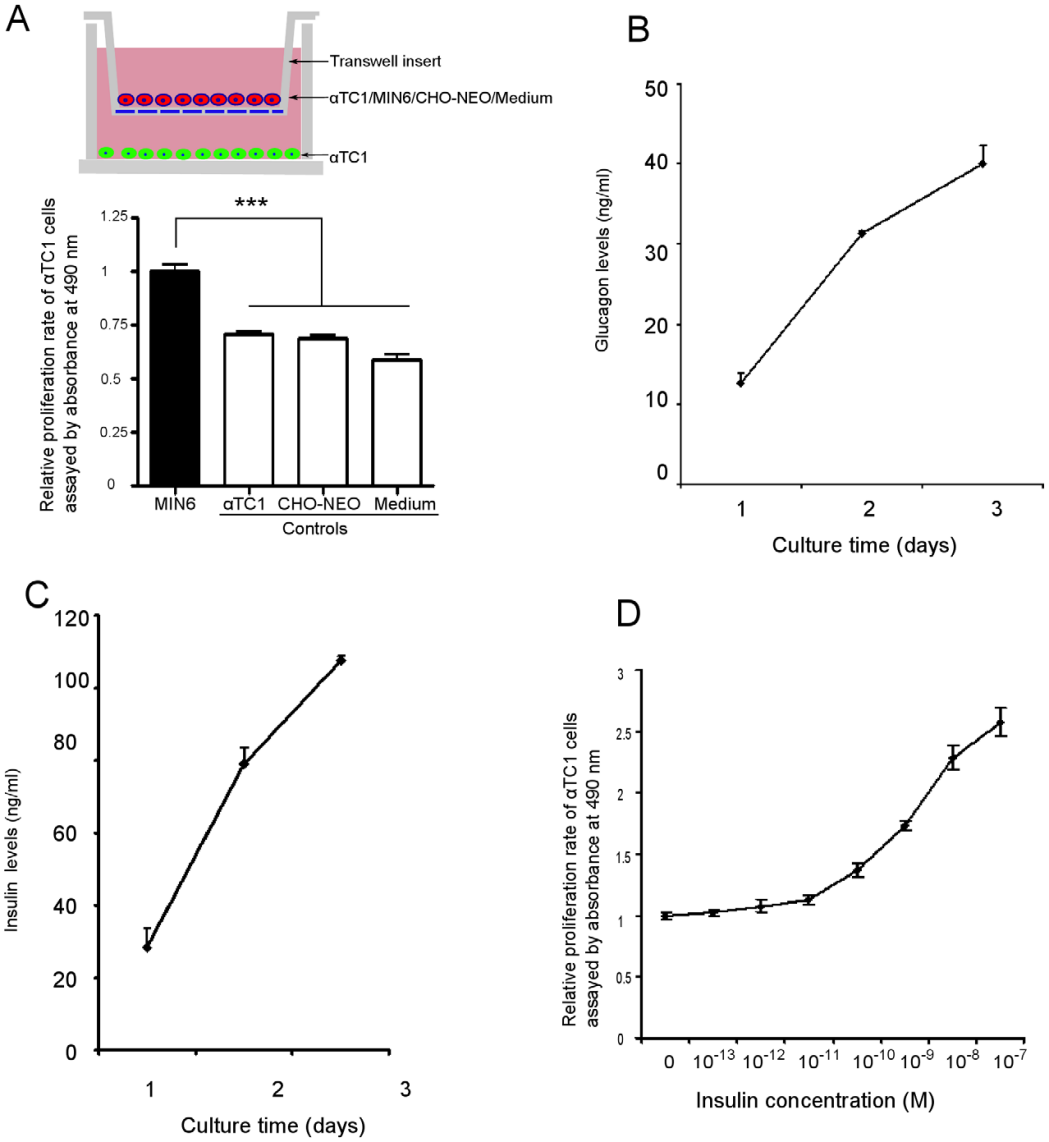
Culturing α cells with MIN6 β cells or insulin increases α-cell proliferation. (A) αTC1 cells were co-cultured with MIN6 β cells (n = 16), αTC1 (n = 16), CHO-NEO (n = 12) and medium (n = 12) in transwell plates, as shown. Results show relative proliferation rates of α-TC1 cells. (B) and (C), glucagon and insulin levels in medium from αTC1 and MIN6 cells, assayed at the times shown. (D) αTC1 cells were treated with insulin for 5 days at the concentration shown. EC50 = 2 nM, n = 6 separate experiments. All data are expressed as means± SEM; ***p<0.0005.

In non-diabetic rodents, the α cells are located on the rim (or mantle) of islets (Figure 1A arrow), while in human pancreatic islets, α cells are additionally interspersed among β cells (Figure 1G for examples) [24]. To mimic free association of endocrine products we used a trans-well co-culture system to culture pancreatic α cells (lower chamber) with β cells and any other cells of interest (upper chamber) (Figure 2A). We found that α-cell proliferation (αTC1) was significantly increased by co-culture with MIN6 cells (a mouse β-cell line) compared with co-culture of α cells with a non-β-cell line (CHO-NEO), αTC1 cells themselves, or medium only (Figure 2A), suggesting that a factor secreted from β cells caused increased α-cell proliferation. α Cells as well as β cells continued to secrete their respective hormones, which accumulated over time in the medium (Figure 2 B,C).

#### 2.2 Insulin increases α-cell proliferation

As insulin is a known trophic factor, especially to β cells [16]–[18], [25], we next studied if it influenced α-cell proliferation. After 5 days of insulin treatment at various concentrations, we found that the α-cell proliferative rate was increased in a dose-dependent manner (EC50 = 2 nM) (Figure 2D), and therefore was most likely a factor in the α-cell proliferation seen when α cells were co-cultured with β-cell lines.

#### 2.3 Insulin up-regulates α-cell proliferation through insulin receptor

**Figure 3:**
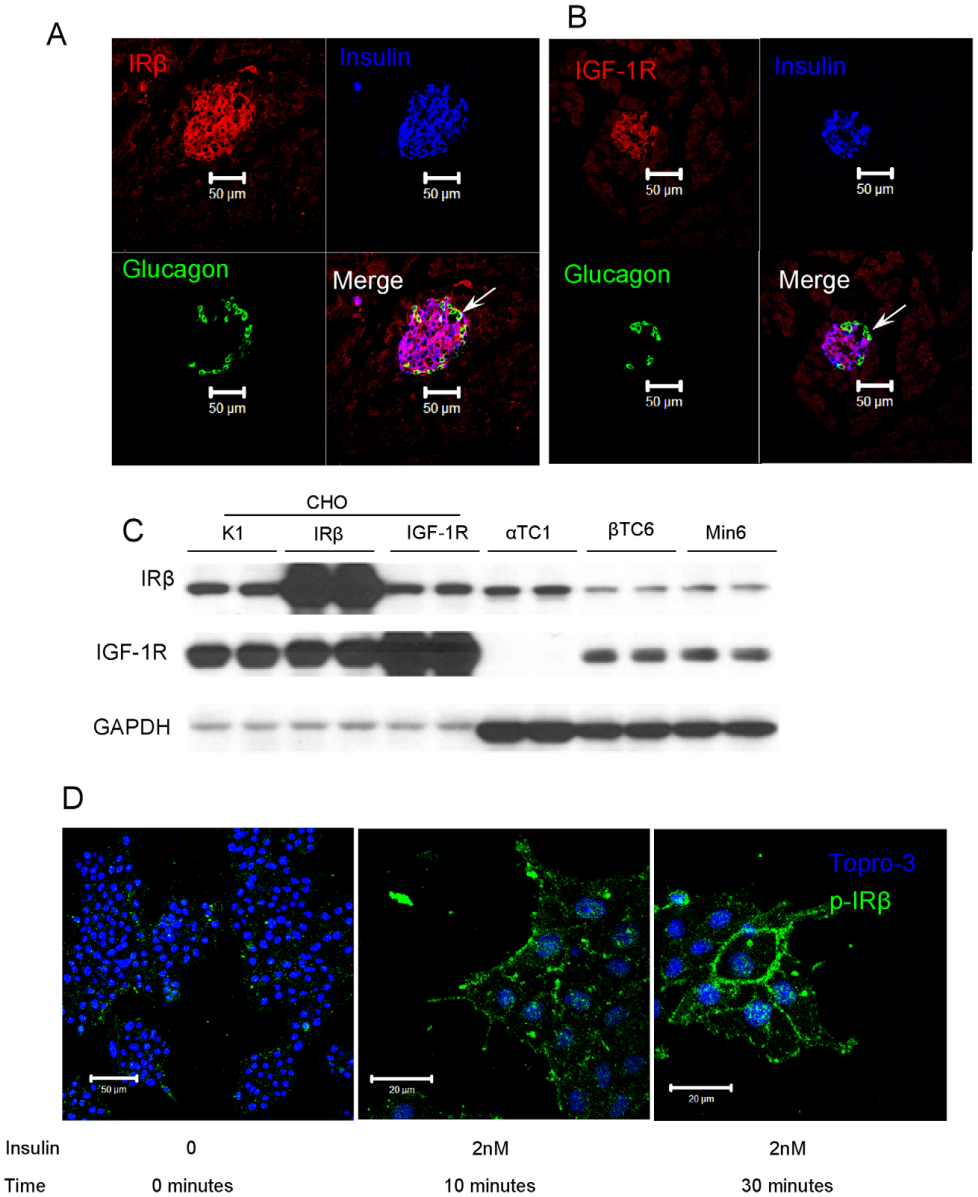
Insulin functions through insulin receptors (IR), not IGF-1 receptors (IGF-1R), in α cells. (A) Immunofluorescent staining of IRβ in a pancreatic islet of a mouse. Merge of IRβ and glucagon is yellow, demonstrating co-localization in α cells (arrow). (B) Immunofluorescent staining of IGF-1R in a pancreatic islet of a non-diabetic mouse. There is no merge of IGF-1R and glucagon (no yellow), demonstrating lack of co-localization (arrow). IGF-1Rs are present on β cells. (C) Western Blot analysis of insulin receptors (IRβ) and IGF-1R in CHO cells, and α-and β-cell lines. IRβ is present in all cell types while IGF-1R is not expressed in α cells (CHO- IRβ are transfected with IR, CHO-IGF-1R are transfected with IGF-1R, CHO-K1 are non-transfected). (D) Immunofluorescence staining of phosphorylated insulin receptors (p-IRβ) in αTC1 cells in response to insulin at the times shown.

It's reported that IRs are expressed on mouse α cells [13]. We confirmed this by immunofluorescence and while mouse islets express IRs on both α and β cells (Figure 3A), IGF-1 receptors (IGF-1Rs) are expressed only on β cells (Figure 3B). We further studied this using western blotting of whole cell extracts from cell lines and confirmed that IRs are expressed in both mouse pancreatic α-cell (αTC1) and β-cell (βTC6 and Min6) lines, but IGF-1Rs are only expressed on β-cell lines (Figure 3C). In agreement with the immunofluorescence data from mouse islets, IGF-1R protein was present in β- but not α-cell lines (Figure 3C). We used CHO cells as positive controls including CHO/K1 (non-transfected CHO cells), CHO/IR (cells that over-express IR) and CHO/IGF-1R (cells that over-express IGF-1R). Thus eliminating IGF-1R on α cells as a candidate insulin receptor. Therefore, we conclude that the effect of insulin on α-cell proliferation is through IRs, not IGF-1Rs.

To prove that the IRs on α cells are functional, we treated αTC1 cells with insulin and observed phosphorylation of IR by immunofluoresence imaging as early as 10 min after insulin treatment, reaching maximum phosphorylation by 30 minutes (Figure 3D).

### 3. Insulin signaling pathway in pancreatic α cells

#### 3.1 Insulin signaling through IR/IRS2/AKT (PKB)

**Figure 4:**
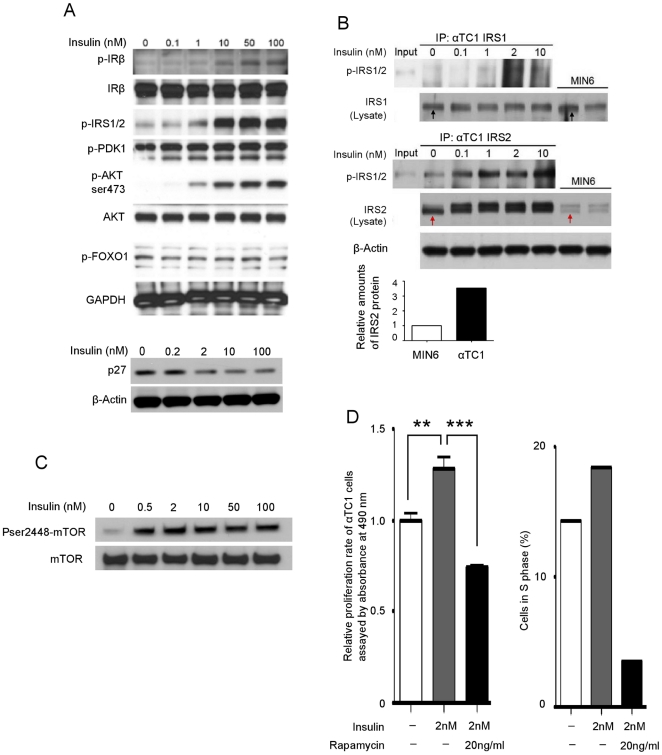
IRs signal through IRS2 and AKT and activate mTOR, resulting in α-cell proliferation. (A) Western blot analysis of insulin receptor signaling molecules in α cells. αTC1 cells were treated with increasing concentration of insulin for 10 minutes and whole cell lysate was used for western blotting. (B) Immunoprecipitation of IRS1 and IRS2 in pancreatic cells. αTC1 cells were treated with increasing concentration of insulin for 10 minutes and whole cell lysates was collected. IRS1 and IRS2 proteins were immunoprecipitated, and immunoblotted with an antibody that recognizes phosphorylated IRS1/2 (Tyr612). Protein levels of IRS1 (black arrows) and IRS2 (red arrows) in αTC1 and MIN6 cells. The densitometry represents relative expression of IRS2 protein levels. (C) αTC1 cells were treated with increasing concentration of insulin for 10 minutes and whole cell lysate was blotted for phosphorylated and total mTOR. (D) α-TC1 cells were treated for 72 hours (left panel) or 24 hours (right panel) with insulin (2 nM) +/− rapamycin (20 mg/ml). The cell proliferation was assessed by both ELISA-based relative proliferation assay (left panel: data are expressed as means ±SEM [n = 6]; ** p<0.005, *** p<0.0005) and by counting the cells in S phase (propidium iodide staining for DNA cell cycle analysis) (right panel).

Since the proliferative effect of insulin on β cells is regulated through the IR/IRS2/PDK1/AKT/FOXO1 signaling pathway [26], we examined this pathway in α cells. We treated αTC1 cells with increasing concentrations of insulin for 10 minutes and whole cell lysate was then used for immunoprecipitation and western blotting. We found that insulin treatment resulted in phosphorylation of IR at Tyr1162/1163, and IRS1/2 at Tyr612 in a concentration dependent manner (Figure 4A). To determine the functional isoforms of IRS in α cells, IRS1 and IRS2 were immunoprecipitated, separated by SDS-PAGE, and immunoblotted with antibodies that specifically recognize phosphorylated IRS1 and 2 (Tyr612). The immunoprecipitation (IP) of IRS1 showed no obvious phosphorylated band (Figure 4B upper panel), but the IP of IRS2 showed an insulin-mediated, dose-dependent increase in IRS2 phosphorylation (Figure 4B, lower panel). Western blotting of whole cell lysates revealed similar amounts of IRS1 in α cells (αTC1) and β cells (MIN6) (Figure 4B, upper panel, black arrows). However, IRS2 protein levels in α cells were four-fold higher than in β cells (Figure 4B, lower panel, and red arrows). Therefore, we conclude that the major insulin receptor signaling molecule in α cells is IRS2.

Multiple growth factor signaling pathways converge at the level of AKT(PKB) activation and further regulate p27, which controls the cell cycle [27]. Similar to β cells [26] we also observed phosphorylation of AKT(S473) and decreasing amounts of p27 (Figure 4A) in response to increasing concentrations of insulin. However, unlike β cells, we found that there was no obvious change in the phosphorylated states of PDK1 or FOXO1 in α cells in response to insulin (Figure 4A). We conclude that α cells have the ability to signal through IR and IRS2, with AKT acting as a key factor in α-cell turnover as well as in β cells, but the down-stream AKT signaling molecules involved in proliferation are not the same as in β cells.

#### 3.2 mTOR signaling in insulin pathway

mTOR, an evolutionarily conserved serine-threonine kinase, interacts with AKT and promotes protein translation and cell growth in response to growth factors [28], [29]. αTC1 cells were treated with insulin for 10 minutes on three separate occasions and lysates were immunoblotted with phosphorylated and total mTOR antibodies. We found that insulin clearly led to phosphorylation of mTOR (Figure 4C).

To further confirm the role of mTOR in insulin-mediated proliferation, we treated α cells with insulin in conjunction with rapamycin, a specific mTOR inhibitor [30]. The cell proliferation was assessed by both ELISA-based relative proliferation assay and by counting the cells in S phase (Propidium iodide staining for DNA cell cycle analysis). We observed that insulin-mediated increases α-cell proliferation was completely abolished in the presence of rapamycin (Figure 4D). Thus, we concluded that insulin up-regulates α-cell proliferation through AKT/mTOR signaling.

### 4. Glucagon regulates pancreatic α-cell proliferation

#### 4.1 Blocking GcgRs decreases α-cell proliferation in vitro

**Figure 5:**
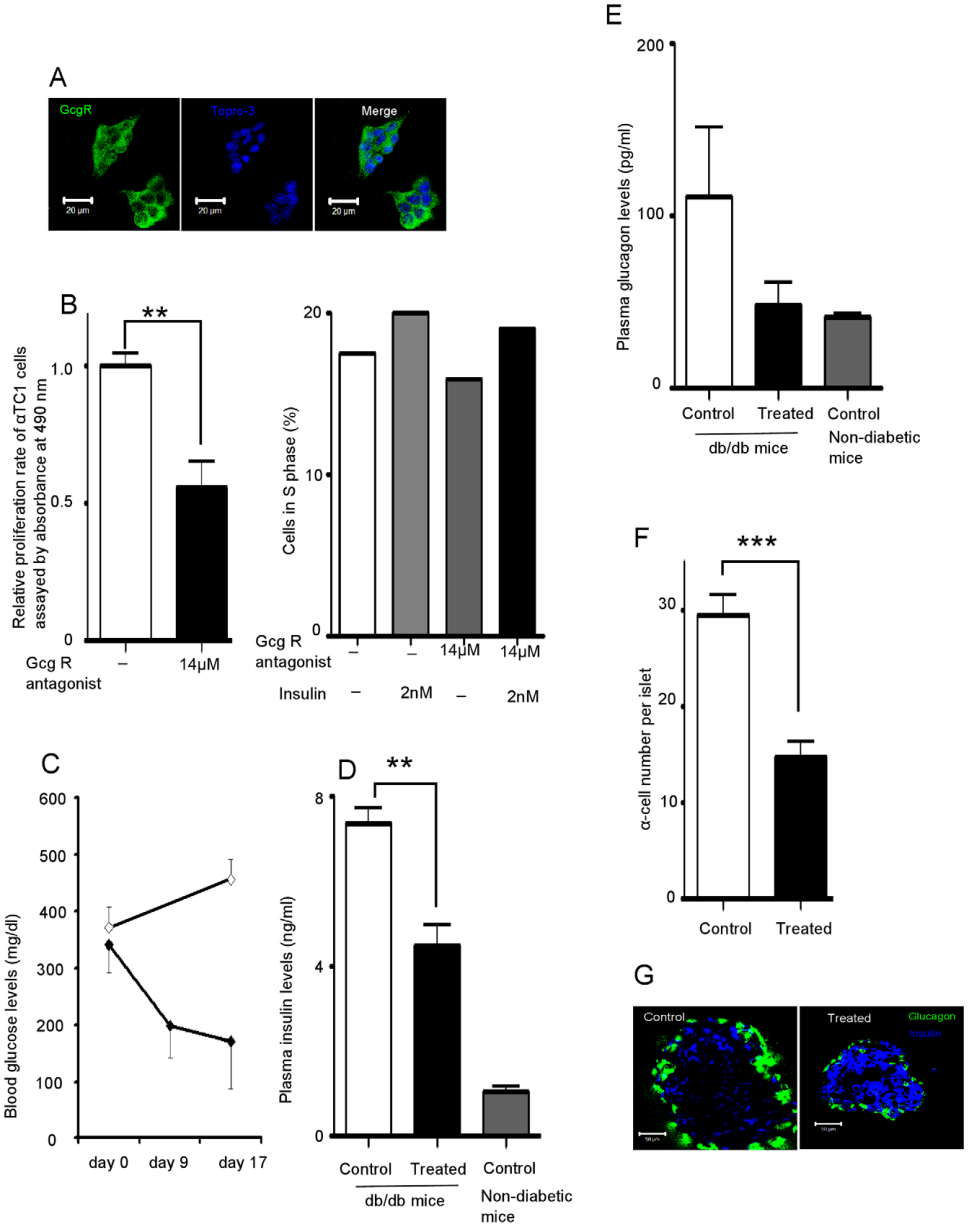
Glucagon receptor (GcgR) antagonism decrease α-cell proliferation. (A) Immunofluorescent staining of GcgR on αTC1 cells. (B) Proliferative response of α cells after 72 hours (left panel) or 24 hours (right panel) of GcgR antagonist II (14 µM) +/− insulin (2 nM) treatment. The cell proliferation was assessed by both ELISA-based relative proliferation assay (left panel) and by counting the cells in S phase (propidium iodide staining for DNA cell cycle analysis) (right panel). (C) db/db mice were treated with GcgR antagonist II for up to 17 days. Blood glucose measurements in non-treated db/db (n = 4, open diamond) and treated db/db (n = 5, closed diamond) mice. (D) Fasting plasma insulin levels of db/db mice measured after 17days of GcgR antagonist treatment. For control db/db mice n = 4, treated db/db mice n = 5, non-diabetic mice n = 7. (E) Fasting plasma glucagon levels of db/db mice measured after 17days of GcgR antagonist II treatment. For control db/db mice n = 4, treated db/db mice n = 5, non-diabetic mice n = 7. (F) α-cell number in islets from db/db mice was counted (control n = 71 islets, treated n = 84 islets). (G) Immunofluorescent staining of glucagon and insulin in frozen sections of pancreata from treated and non-treated db/db mice. Data are expressed as means ±SEM (n = 6); ** p<0.005, *** p<0.0005.

In the early stages of T2DM, insulin levels are elevated due to insulin resistance, so it is possible that on-going, sustained elevated intra-islet insulin levels are conducive to α-cell proliferation, especially if α-cell proliferative brakes are dysfunctional, and it is also possible that as glucagon secretion begins to rise that it serves to stimulate α-cell proliferation. Blocking GcgRs has been reported to improve glucose homeostasis [19]–[21], glucagon stimulates its own release and GcgRs are reported to be present on α cells [10]. Immunofluorescent staining confirmed the presence of GcgR on αTC1 cells (Figure 5A) and after 72 hours of treatment with GcgR antagonist (14 µM) so as to block the effects of the endogenously secreted glucagon, we observed a 44% decrease in αTC1 cell proliferation compared with the untreated group (Figure 5B left panel). We further confirmed this by counting the cells in S phase, and found that 2 nM of insulin still increased αTC1 cell proliferation even in the presence of GcgR antagonist (Figure 5B right panel).

#### 4.2 Blocking GcgRs decreases α-cell number and improves glucose homeostasis in diabetic mice

We next treated db/db mice (2 months old, male, N = 4 mice per group) with a GcgR antagonist or vehicle delivered by gavage daily, for 17 days. Compared to day 0, we observed a 50% decrease of plasma glucose levels while in vehicle-treated animals blood glucose levels continued to increase (Figure 5C). Compared with the vehicle-treated group, we observed a 39% decrease of insulin (Figure 5D) and a 56% decrease of glucagon plasma levels (Figure 5E) and a 50% decrease in α-cell number (Figure 5F). Immunofluorescent staining also showed increased intra-islet insulin in the antagonist-treated mice (most likely due to less degranulation of β cells) and smaller α cells (Figure 5G).

## Discussion

Our results firmly establish that elevated levels of glucagon and increased pancreatic α-cell number are two principle factors in the progression of diabetes and, in fact, compounding the problem, α-cell numbers continue to increase with worsening diabetes in db/db mice. As the leptin receptor is inactivated in db/db mice leptin cannot be directly impacting α-cell number, though it is indirectly influencing α-cell number by causing diabetes. There is human data that concur with our findings of increased α-cell numbers and increased α- to β-cell ratios in T2DM [15]. Therefore, we conclude that increased plasma glucagon levels in T2DM are accompanied by increased α-cell numbers.

The molecular mechanisms underlying α-cell proliferation in diabetes have not been studied. Insulin signaling in α cells is involved in suppression of glucagon secretion and it was very recently reported in mice that knockout of IRs in α cells (αIRKO mice) leads to a decrease in α-cell mass, which becomes more evident with age [13]. Our results support the notion that insulin could potentially influence not only α-cell function but also turnover. There is precedent for this notion in islets because insulin regulates pancreatic β-cell proliferation through IR/IRS2/PDK1/AKT/FOXO1 pathway and its knockout in mice leads to reduced β-cell mass [26]. Our results indicate that AKT might be a key molecule involved in insulin modulation of α-cell proliferation, but neither PDK1 nor FOXO1 are involved. It has been reported that mTOR is required for AKT-dependent cell growth in human retinal cells [31] and our data show that insulin activates mTOR in α cells. Furthermore, in the presence of rapamycin, an mTOR inhibitor, the effect of insulin on α-cell proliferation and mTOR phosphorylation is completely abolished.

In insulin resistant states, β cells attempt to compensate for the resistance by secreting more insulin. In pancreatic islets, this results in higher intra-islet insulin concentration than normal, resulting, in turn, in increased IR signaling. This initially would be predicted to cause a decrease in glucagon secretion but eventually, insulin resistance must occur to the suppressant effect on secretion, and ultimately dysregulation of glucagon secretion. We hypothesize that, in the early stages of insulin resistance, there are counter-regulatory systems to the trophic effects of insulin on α-cell proliferation. But as insulin resistance increases, the counter-regulatory measures within islets may become dysfunctional or overridden by high insulin levels, leading to increased α-cell turnover and ultimately more glucagon released into the circulation. This will worsen insulin resistance in the liver, leading to increased, non-suppressible gluconeogenesis and increased fasting blood glucose. Moreover, glucagon, through its G protein-coupled receptor on β cells, enhances insulin secretion and β-cell mass (8), an effect that is likely to be of particular importance in human islets because of the unique β-/α-cell proximity: so increasing glucagon secretion could be another compensatory attempt by the body to increase insulin secretion in insulin resistant states.

It is probable that in T2DM α cells become resistant only to the inhibitory effects of insulin on glucagon secretion and not to the trophic effects. There is precedent for selective insulin resistance. In the liver, the FOXO1 pathway becomes insulin resistant in obese and diabetic states and this results in decreased glucose uptake and continuing gluconeogenesis; and yet, despite this, insulin sensitivity is maintained in the SREBP-1c pathway, which leads to increased fatty acid synthesis and excess triglyceride secretion from hepatocytes that further ultimately worsen insulin resistance in muscle [32], [33]. The inhibitory effects of insulin on glucagon secretion are FOXO1-dependent because knockdown of the IRs in α cells by siRNA led to markedly reduced pFOXO1 and increased glucagon secretion [13] and FOXO1 silencing abolished the acute regulation by insulin of glucagon secretion [34]. Analogous to the situation in hepatocytes, the α-cell FOXO1 pathway is probably insulin-resistant in T2DM. And as we found that the trophic effects of insulin in α cells are not mediated by FOXO1, it is reasonable to conclude that the effects of insulin on α-cell proliferation are favored by the high intra-islet insulin levels, while the suppressant effects on glucagon secretion are obviously abrogated as plasma glucose rises in T2DM. It would be expected that there may be increased amounts of other products of the proglucagon molecule, GLP-1 for example, circulating in diabetic conditions. However, GLP-1 levels are not elevated in type 2 diabetic conditions [35] and GLP-1 receptor seems to be exclusive in islets to β cells [35], [36]. Therefore, we do not think a proglucagon product is the primary proliferative factor to α cells.

Our results show that blocking the glucagon receptor decreased blood glucose, as expected, but it also decreased α-cell proliferation in both mice and αTC1 cells, leading us to conclude that glucagon itself has a direct trophic effects on α cells, just as it does in β cells. Furthermore, when pancreatic α-cell number was decreased in half in db/db mice by a glucagon receptor antagonist, plasma glucagon levels were significantly decreased, again drawing a firm connection between increased α-cell number and secretion. Interestingly, plasma levels of insulin dropped and β-cell degranulation was lessened, most likely reflecting decreased insulin secretion and decreased intra-islet insulin, which would also lessen the trophic effects of insulin on α cells.

It should be borne in mind that the effects of a glucagon antagonist would have different effects in non-diabetic compared to diabetic mice. In non-diabetic mice, an antagonist causes low blood glucose levels, which lead to a sympathetic nervous system response and α-cell proliferation as the animals attempt to compensate for neuroglucopenia [19]. In diabetic mice, no such adrenergic response would be expected if blood glucose is not lowered below normal.

Lowering plasma glucagon represents an attractive therapeutic approach for T2DM, and recent success in this field has generated considerable enthusiasm [19]. Blocking of glucagon receptors in diabetic mice led to significantly improved blood glucose control and decreased plasma insulin levels. This likely resulted from alleviation of the insulin resistance and a decrease in the stimulus to insulin secretion by glucagon. Thus, the burden on β cells is reduced, which results in, as showed in our data, less degranulation of β cells.

In summary, we provide evidence that blocking mTOR prevents α-cell proliferation. The mTOR pathway plays a crucial role in tumorigenesis [37], [38] and rapamycin and the other inhibitors of mTOR have been investigated as anti-cancer medications in recent years [39]–[42]. Very interestingly, rapamycin just recently was shown to increase life-span in mice even when given late in life [43] and mTOR inhibitors (or rapalogs that do not have the immunosuppressant effects of rapamycin), are now being explored by companies to extend human life [44]. Based on our data, such analogs might be worth exploring in treating T2DM. We also provide evidence that blocking glucagon receptors in T2DM may not only lessen glucagon-mediated gluconeogenesis, but may be beneficial to β cells.
